# Bibliographical

**Published:** 1887-03

**Authors:** 


### EDITORIAL.

### BIBLIOGRAPHICAL.

### "The Microscopic Structure of the Human Tooth, together with some Unusual and Irregular Forms of Teeth.

BY CHARLES H. STOWELL, M.D., F. R. M. S., PROFESSOR OF HISTOLOGY AND MICROSCOPY IN THE UNIVERSITY OF MICHIGAN; AUTHOR OF STUDENTS' MANUAL OF HISTOLOGY, MICROSCOPICAL DIAGNOSIS, LATE EDITOR OF THE MICROSCOPE, ETC., ANN ARBOR, MICH.

The author, in the preface, says "the purpose of this work is two-fold: First, to give to the dental profession plans and definite statements concerning the minute structure of the human tooth. Second, to place on record some of the specimens from the unique collections of Professors Ford and Taft. To accomplish the first, a careful study has been made of both young and old teeth, after methods described in the general text. All the specimens were drawn in India ink by the author, and reproduced by the engraver, in such a manner that the writer alone is responsible for the accuracy of the illustrations. It is believed they fairly represent what is known to day on this subject. It cannot be expected that everything said in the following pages will agree with the opinions held by all workers. They are the opinions of the writer, and as such must go for what they are worth. To accomplish the second object of the work, it was only necessary to make accurate drawings of the specimens from these collections."

Such a work as this has not been attempted heretofore in this country, so far as we are aware. The engravings are finely executed upon heavy card-board, 11x16 inches, and every one, from natural specimens, was drawn by Professor Stowell, and in such a manner as to be beyond criticism. A new and very valuable feature has been introduced in what is called "The Working Diagram." Of it, the author says, "By referring to this diagram, the position of any of the structures of a tooth can be easily ascertained. It will also enable the reader to locate the various structures on the following pages." This, so far as we know, is a new idea, wholly original with Dr. S. Its value is at once apparent to every observer. The diagrams showing the structure of the different tooth-tissues is taken from sections under the microscope, so that they may be regarded, true to nature. Not one is a copy from any other drawing or engraving.

We will call attention to the transverse section of the root of a biscupid, the complex structural characteristics of which are shown most beautifully. This, we think, was never so perfectly done before. The diagram exhibiting the blood vessels of the pulp is a marvelous presentation of the structure of this organ. The preparation of these sections with such perfection is indeed a wonder, and the drawing of the preparations is a remarkable exhibition of skill. So perfect a specimen has never before been presented, the arteries, veins, and capillaries all being fully portrayed. The section of the root parallel to the dental canals exhibits most clearly and beautifully the structure of the dentine, the granular layer, and the cementum. It is given larger and more in detail than in any similar illustrations we have ever seen. The plate showing the living parts of the tooth is very clear and understandable, showing the odontoblast, the linings and contents of the "dentinal fiber of the tubuli;" also the living matter of the lucanæ of the cement. The three plates, showing seventy-five figures of dental anomalies, are well worth special attention and study. A large variety in form, and size, and number of roots, and other peculiar characteristics are presented. Each and every one presented is an object worthy of study, and, with the explanatory notes, will be fully understood. In addition to the illustrations here referred to, there are fourteen pages of text, same size as plates, descriptive of the microscopic structure of the human teeth. The dental enamel, cement, and tooth-pulp, each receive special descriptive attention. Three pages are given, descriptive of the methods of examining the teeth; these, though brief, are very instructive. The work, upon the whole, is a valuable one to every one who wishes to study the histological structure of the teeth thoroughly. It should be in the hands of every dentist, in the first place, for his own information and satisfaction, and with it he may do much in the way of giving information in reference to the teeth, to intelligent patients. Such a work as this has long been needed, and it should be in the hands of every dentist. It is published by, and can be obtained of, the author, Prof. Charles H. Stowell, M.D., Ann Arbor, Michigan. Price $6.

